# Methodology of an exercise intervention program using social incentives and gamification for obese children

**DOI:** 10.1186/s12889-019-6992-x

**Published:** 2019-06-03

**Authors:** Yue Fang, Yunsheng Ma, Dandan Mo, Shunxing Zhang, Mi Xiang, Zhiruo Zhang

**Affiliations:** 1 School of Public Health, Shanghai Jiao Tong University, Shanghai, 200025 China; 2 Division of Preventive and Behavioral Medicine, Department of Medicine, University of Massachusetts Medical School, Worcester, MA 01655 USA; 3 Scarsdale High School, Scarsdale, NY 10583 USA

**Keywords:** Exercise, Social incentive, Gamification, WeChat, Metabolic health, Healthy behaviors

## Abstract

**Background:**

Traditional exercise [supervised exercise (SE)] intervention has been proved to be one of the most effective ways to improve metabolic health. However, most exercise interventions were on a high-cost and small scale, moreover lacking of the long-term effect due to low engagement. On the other hand, it was noteworthy that gamification and social incentives were promising strategies to increase engagement and sustain exercise interventions effects; as well as mobile technologies such as WeChat also can provide an appropriate platform to deploy interventions on a broader, low-cost scale. Thus, we aim to develop a novel exercise intervention (‘S&G exercise intervention’) that combines SE intervention with gamification and social incentives design through WeChat, with the aim of improving metabolic health and poor behaviors among overweight and obesity children.

**Methods:**

We propose a randomized controlled trial of a ‘S&G exercise intervention’ among 420 overweight and obese children who have at least one marker of metabolic syndrome. Children will be randomized to control or intervention group in a 1:1 ratio. The exercise intervention package includes intervention designs based on integrated social incentives and gamification theory, involving targeted essential volume and intensity of activity (skipping rope) as well as monitoring daily information and providing health advice by WeChat. Participants will undertake assessments at baseline, at end of intervention period, in the follow-up time at months 3,6,12. The primary outcome is outcome of metabolic health. Secondary outcomes include behavioral (e.g., diary physical activity, diet) and anthropometric measures (e.g., body fat rate and muscle mass).

**Discussions:**

This will be the first study to design an exercise intervention model that combines traditional supervised exercise (SE) intervention with gamification and social incentives theory through WeChat. We believed that this study could explore a low-cost, easy-to-popularize, and effective exercise intervention model for improving metabolic health and promote healthy among obese children. Furthermore, it will also provide important evidence for guidelines to prevent and improve metabolic health and health behaviors.

**Trial registration:**

10-04-2019;Registration number: ChiCTR1900022396.

## Background

Childhood obesity is correlated with cardiovascular disease (CVD) and type 2 diabetes in adulthood [1–3]. The worldwide prevalence of childhood obesity has been steadily increasing in the past decades [4, 5]. China has now joined this world epidemic, a recent survey suggested that mean prevalence of overweight and obesity increased from 5.3% (5.2–5.4) in 1995 to 20.5% (20.4–20.7) in 2014 [6]. Moreover, a Chinese study reported that in 2010–2015, the age-adjusted prevalence of overweight and obesity among boys increased from 21.2 to 31.7% and from 10.6 to 16.9% among girls [7]. It has been proved that obese children are more likely to have metabolic diseases than his/her non-obese peers [8]. Indeed, up to 90% of overweight adolescents had at least one marker of metabolic syndrome and 56% had two markers [3]. Moreover, the risk of metabolic diseases in adults is formed from childhood [9], and some studies has demonstrated that metabolic abnormality in childhood obesity may be a significant predictor to CVD and other chronic diseases in adulthood [10, 11]. Therefore, it is important for obese children to improve metabolic health in early childhood.

There is strong evidence that exercise, especially traditional supervised exercise (SE), is one of the effective ways to improve metabolic disease risk factors [12, 13]. As the traditional supervised exercise (SE) ensure intensity and dose of physical activity. Indeed, the importance of activity intensity and dose has been also emphasized in several systematic reviews [13–15].

A systematic review has found that only moderate and high intensity exercise can improve health outcomes such as obesity and elevated blood glucose level, rather than low intensity exercise [16]. Therefore, it has even been demonstrated that SE interventions is even more effective than daily physical activity (PA) intervention in improving outcomes including metabolic biomarkers [16]. However, previous SE intervention was mainly supervised by health professionals, resulting in high cost and difficult to translate into large scale [16–18]. In addition, most SE interventions are lack of motivation and interest, because it specified repetitive exercise content or form. Which also makes it difficult for participants to develop the exercise habit and keep on when the intervention stopped, so that the long-term effect of interventions is questionable [19, 20].

The rapidly expanding availability of mobile technologies such as wearable devices and smartphones makes up limitations above and provides an opportunity to deploy interventions on a broader, low-cost scale [21]. In particularly, WeChat (the Chinese version is Weixin), as a representative form of modern messaging software, reaching 1058 million active users, has been used to healthy behavior modifications and already shown potential impacts [22, 23]. It has been demonstrated that using the WeChat app for follow-up visit was time-effective, cost-effective, and convenient [24].

Besides, latest researches have highlighted two features important for behavior change: (i) Social Incentive; (ii) Gamification. Social incentives, or the influences that motivate individuals to adjust their behaviors based on social networks, is ubiquitous and is an effective way to increase engagement and stimulate the long-term health behavior changes [21, 25]. There is a recent call for studies on the potential uses of social engagement strategies in promoting health published by the New England Journal of Medicine [25]. The latest randomized systematic review also suggested that the intervention group with increased social incentives such as peer support and mutual encouragement was more significant change than the control group in healthy behaviors [26].

Additionally, gamification, the application of game design elements such as points and levels in non-game contexts, in order to enhance the interest of context, is increasingly being used to promote changes in healthy behaviors, especially PA [21, 27]. A randomized controlled trial published in JAMA Internal Medicine tested the effectiveness of gamification-based design in improving and maintaining healthy behavior [28]. Besides, gamification-based design not only improve the healthy behaviors after intervention, it has also a positive effect on long-term maintenance.

Social incentives in combination with gamification interventions can make up the limitation of SE intervention. However, so far little research has done to combine SE intervention with gamification and social incentives to improve metabolic health.

Therefore, we propose a randomized controlled trial of a ‘S&G exercise intervention package’ among obese and overweight children. The intervention package includes intervention designs based on integrated social incentives and gamification theory, involving targeted essential volume and intensity of activity (skipping rope) as well as monitoring daily information and providing healthy advice by WeChat.

The aims of this study are twofold. First, the randomized controlled trial targeted obese and overweight children, is to assess the effectiveness of a ‘S&G exercise intervention’ to improve metabolic health. The second aim is to test the effect of this innovative exercise intervention on healthy behaviors changes such as PA, diet, sleep health and screen media use, which are significantly associated with healthy behaviors and health status in their adulthood. We hypothesize that the intervention will improve metabolic health and unhealthy behaviors in obese children. Furthermore, it will make a significant contribution to childhood health research and practice by developing a novel intervention model to promote metabolic health and healthy behaviors among obese children.

## Methods/design

### Study design

**Fig. 1:**
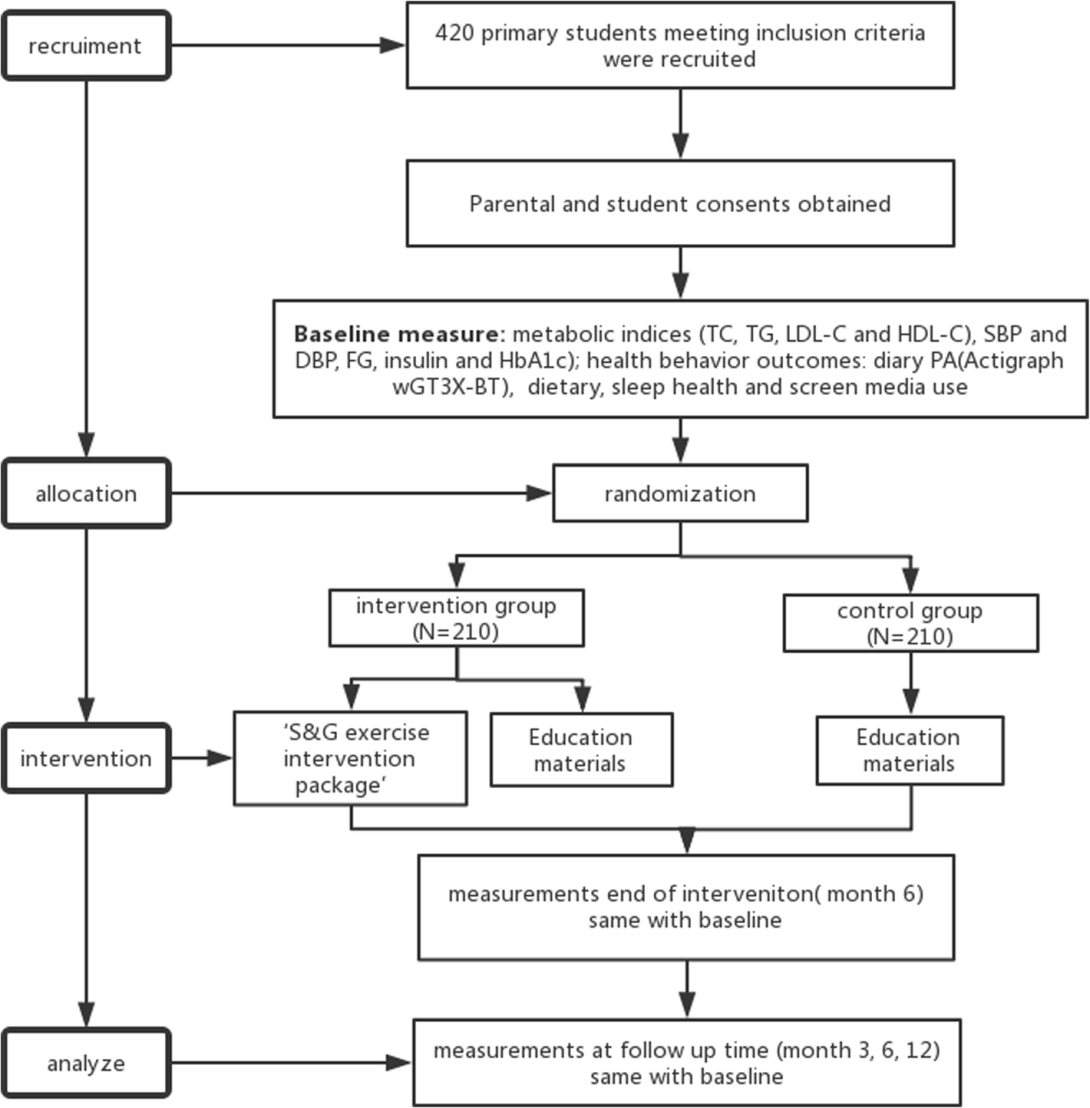
Study design flow chart. *‘S&G exercise intervention package’* (deliver via WeChat and Actigraph wGT3X-BT;). Social incentive intervention: Divide the intervention group into 30 teams; Organize a kick-off meeting; Report daily information within Teams; Others: Peer support; Accountability; Team discussion. Gamification intervention: Members get points from different types of game activities; Use performance as punishment for members who fail to complete tasks; Rank, badge and reward according to points; Held a skipping competition online

This protocol describes the setting of a 6-months intervention period and 12-months follow-up period assessing the impact of a ‘S&G exercise intervention package’ (targeted an intervention integrated social incentive theory and gamification theory with a smart phone app as support) on improving metabolic health in overweight and obese children. Participants were randomly assigned to either an intervention or a control group. The complete process is outlined in Fig. 1.

### Ethics approval

The study protocol was approved by the Ethics Committee of Shanghai Jiaotong University School of Medicine (SJUPN- 201813). This research is supported by the National Natural Science Foundation of China (71804110).

### Sample size consideration

Based on the relative studies on exercise intervention with obese children [29], the sample size required for each group was estimated to be about 175 with a power of 80%, an α of 0.05, and assumed an effect size of 0.30. Therefore, a total of 350 children are need. To account for 20% loss-to-follow-up, a total of 420 children will be recruited.

### Recruitment and consent

Participants will be recruited randomly from primary schools in Shanghai. We will send invitation letters to primary schools. Before confirming their participation in the study, the study team will arrange a briefing session with the representatives of both school and parent interesting in participating to outline the study protocol. After confirmation of their participation, a total of six schools will be randomly selected.

Eligible individuals, accompanied by their parents, will be provided with consent forms including blood sample collection and physical examination before participating in the study.

### Inclusion criteria

Children are eligible for the study participation if they meet the following inclusion criteria.

1. overweight or obese: according to the China school age children and adolescents overweight and obese BMI screening standard published by the Working Group on Obesity in China (WGOC) (see Table 1) [30]
2. abnormal metabolic indicators: having any one of the following indicators defined mainly according to the criteria of the International Diabetes Federation (IDF 2007) adapted for children and adolescents [31]
3. fasting glucose (FG) ≥ 5.6 mmol/L
4. systolic blood pressure (SBP) ≥ 130 mmHg and/or diastolic blood pressure (DBP) ≥ 85 mmHg or was diagnosed with hypertension
5. HDL-cholesterol < 1.03 mmol/L, HDL cholesterol (boy) < 1.03 mmol/L, HDL cholesterol (girl) < 1.29 mmol/L
6. triglycerides (TG) ≥ 1.7 mmol/L
7. elevated HbA1c defined as 5.7–6.4% according to the 2010 American Diabetes Association (ADA) criteria [32]

### Exclusion criteria

Participants are excluded if they are already participating in an exercise program, have been told not to exercise by a physician, have secondary obesity or metabolic abnormalities caused by hypothyroidism, injury of hypothalamus and so on, take medication that affects metabolism within 3 months before intervention, or if there is reason that participation is unsafe or infeasible for the individual.

### Randomization

The study will use stratified random sampling, and the random sequence of primary schools will be computer generated. Specifically, it stratified by school district, grades and size in each center. All of the children in the selected primary schools of different grades were invited to participate in the study.

After both parents and children written informed consent have been obtained, it will be allocated into either an intervention or control group in a 1:1 ratio. Neither the study team nor the participant knew the group allocation prior to the start of the intervention program.

### Control group

The children in the control group will not receive a ‘S&G exercise intervention package’ but will receive and wear wearable devices-Actigraph wGT3X-BT (Pensa- cola, FL) accelerometers. Research assistants will send helpful nutritional and exercise tips via WeChat message.

### Intervention

We used WeChat as the social media platform to carry out the intervention. A WeChat group is created for each group by the investigators. The intervention group will receive skipping rope plus the ‘gamification exercise package’. Data will be collected five times: at baseline, at end of intervention period, in the follow-up time at months 3,6,12.

### Nominated helpers-parents

Children can nominate their parents to be an official helper. The official helper will represent children and report daily information to the WeChat group. Children lack willpower and this way also may have potential benefits for children via increased support and motivation.

### Exercise protocol

There is strong scientific evidence that exercise substantially have well-documented health benefits including reducing metabolic disease risk and body fatness [12]. To achieve these benefits, WHO recommends that the level of exercise for a 5–17-year old is 60 min per day at moderate-to-vigorous intensity level.

The skipping rope was chosen as the main form of exercise intervention because it adopts the high popularity and acceptance among Chinese primary school students, as it was set to be tested by The State Education Bureau of China. Moreover, the skipping rope meet the WHO exercise recommendation for children and benefit for child growth. Additionally, considering the condition that pupil in shanghai primary school have physical education class which lasts 45 min every day, so we decide to set the volume of exercise as rope skipping for 1000 (approximately 10–15 min) per day.

Previous review indicate that no less than 6 months of intervention was the most effective duration, so the project is consisting of a 6-month intervention period and a 12-month follow-up period.

### ‘S&G exercise intervention package’

The intervention lasts for six months. It will be delivered via a smartphone app (WeChat) and an Actigraph wGT3X-BT. Several behavioral change theories will guide the intervention, including Social Incentive Theory and Gamification Theory.

### Social incentive intervention design

Previous research has found that a combination of individuals and teams could increase participants’ engagement [33]. Peer support, accountability and team discussion also has been proved to enhance social incentives [34]. Thus the intervention will add these features to promote enduring involvement:

1. Grouping: In order to enhance social incentives, WeChat groups are established respectively for the intervention group and the control group by the investigators. The intervention group will be divided into 30 teams involving 6–7 members according to BMI, age and sex. Because a pilot study has showed that one group within 5–6 members had the best partner interaction [35].
2. A kick-off meeting: After randomization, a kick-off meeting will be organized by the investigators and all participants in the intervention group will be asked to attend. In the meeting, the investigators will introduce the intervention protocol and ensure teammates fully understand what they need to do. Then, each team will perform several interactions to foster familiarity and cohesion among their teammates, as well as elect their team leader. A kick-off meeting can increase effective and positive interaction in social groups and promote engagement as well as motivation [36].
3. Daily Report within Teams: Parents urge the children to skip rope every day and report the daily information (include: data recorded by Actigraph wGT3X-BT; ‘whether the number of rope skipping reach the standard’) to their team WeChat group before 22:30 PM on the day.
4. Peer Support: Each day teammates (the family who represent child) develop at least one interaction in the WeChat group. Praising teammates who reach the standard and encourage those who don’t reach (e.g., ‘Doing well and keep going.’) are also required. Criticism and ridicule, which might cause negative reactions, are not allowed.
5. Accountability: Team leader responsibility system is implemented in the group. The team leader is responsible for reminding their members to complete daily task and send “PS” (the abbreviation of peer support). The team leader rotates once every month for fairness.
6. Team discussion: Healthy tips will be posted by the study team in each group every Friday. Participants will be guided to read it and communicate with teammate on their gains as well as barriers of the program.

### Gamification intervention design

**Fig. 2:**
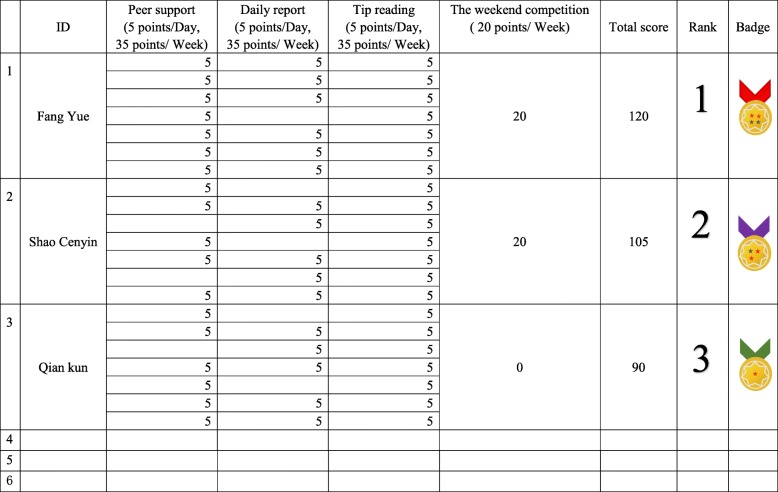
Template for the points leaderboard. Ranking was determined by total points. All participants could access the leaderboard to see their ranking and badges in comparison with all others

**Fig. 3:**
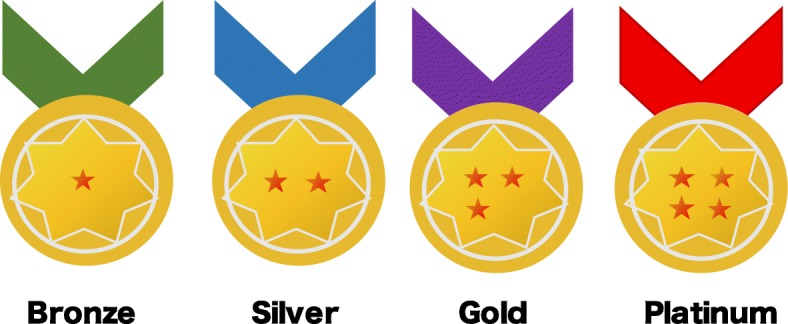
The example of the badges

Points, ranking, badges, punishment and reward are typical elements of gamification, and have been confirmed as effective techniques to improve the interest, incentive and purposiveness of non-game programs [27, 37]. They will be used within the intervention, and to encourage frequent use and support ongoing engagement of both participants and helpers. The detail elements are as follows:

1. Points: Each week, the members and teams will be rewarded for the successful accomplishment of specified activities. The activity consists of four parts, including peer support, daily report, the weekend competition and tip reading. Different types of activities will result in different pinots. For example, if an individual reports daily information on time, he or she would earn 5 points per day; reading tips earn 5 points per day. If they take part in the competition on weekend, earn 20 points. The points leaderboard see Fig. 2.
2. Ranking: Each week, every member in a team and all teams will be ranked according to the final points, and ranking will be announced every Sunday.
3. Badges: Different points set different badges. It is suitable for both teams and individuals. All will begin in the bronze level. Higher levels include silver, gold, and platinum. The example sees Fig. 3. The higher the point, the higher the level. In addition, the advanced individuals award the certificates to motivate and encourage all participants to compete with one another.
4. Punishment: If a member does not finish daily task (or forget to do), the person will be asked to do some performances in WeChat group such as posting a voice messages of song, a standup comedy, or a dance video. Besides, if team leader fails to urge teammate on time, the leader also need to perform. Utilizing performance as punishment is an original design and it could not only urge participants to follow the rules yet avoid embarrassment, but also improving compliance.
5. Rewards: Once a month, each member of the team that accumulated the highest points will be rewarded with a diploma. Besides, the team which get high point will be rewarded corresponding badges as their WeChat group name and it will be showed in a leaderboard.
6. Competition: Every weekend, each team will hold a skipping competition online (or offline for their convenient) in order to stimulate the teammates to engage in teamwork. The content is the number of rope skipping in one minute. All members will participate in the competition in principle, and the one who does best or the team which get the highest score at rope skipping will be rewarded with a virtual certificate, respectively. In addition, each team will calculate the per capita number for ranking.

### Message and feedback

Participants have regular contact with the study team each week via WeChat message to ascertain if there are any difficulties such as changing exercise habits and how they are uploading the information. These messages are structured and standardized, and all participants in the intervention group receive the same message. The contents of these messages are composed using motivational language and feedback is encouraged which is recorded to help assess compliance with the intervention.

### Outcome measures and measurement procedures

A full list of measurable outcomes is presented in Table 1. Participants will be assessed at baseline, at end of intervention period, in the follow-up time at months 3,6,12. Assessment will be undertaken by an experienced research health workers from primary school and Shanghai children’s hospital. All staff involved in data collection will be given training in study procedures before the study.

**Table 1:** Summary of outcome measures

Outcome	Item	Device
*Primary outcomes*		
Metabolic indices	Fasting glucose, triglyceride, total cholesterol, HDL-C, LDL-C, insulin, HbA1c and blood pressure	Blood analyze and blood pressure measurement
*Secondary outcomes*		
Healthy behavior	Diary PA	Accelerometer (Actigraph wGT3X-BT; 7 consecutive days, 24 h/day); IPAQ-A questionnaire
	Diet	DSQ (Dietary Screener Questionnaire)
	Sleep health	Accelerometer (Actigraph wGT3X-BT; 7 consecutive days, 24 h/day); PSQI questionnaire
	Screen media use	Self-designed questionnaire
Anthropometric	BMI, height, weight, waist circumference, body fat rate and muscle mass	Physical examination

*DSQ* Dietary Screener Questionnaire, *PSQI* The Pittsburgh Sleep Quality Index, *IPAQ-A* International Physical Activity Questionnaire

1. Biomarkers: We will use a basic panel of tests to evaluate a comprehensive metabolic profile using overnight fasting blood draw. After an overnight fast of at least 10 h, 5 ml venous blood samples will be taken from the antecubital vein and collected for measuring biochemical parameters [38]. All biochemical analyses on blood will be carried out at a validated biomedical analyses laboratory. Fasting glucose, triglyceride, total cholesterol, HDL-C, and LDL-C were analyzed. Insulin resistance will be estimated using the homeostasis model assessment of insulin resistance (HOMA-IR) = (fasting insulin (FI) x fasting glucose (FG)/22.5, and insulin β cell base secretion index (HOMA-β) = 20 *FI/(FG-3.5) [39].
2. Anthropometric measures: We will measure height, weight, waist circumference and muscle mass via Tanita BC-420 body composition analyzer. BMI (kg/m^2^) is the standard diagnostic tool to assess obesity and overweight in the children and will be calculated as body weight (kg) divided by the square of height (m). Height will be measured using a Seca Leicester Height Measuring Stadiometer, with participant facing forward, wearing no shoes and with their head in the Frankfort Plane (parallel to the floor). Measurements will be recorded once, in cm, to one decimal point. Weight will be measured, in the absence of shoes, using Tanita HD 352 High-Capacity Low-Profile Electronic Weighing Scales. Scales will be calibrated before first use. Weight will be recorded once, in kg, to one decimal point. Waist circumference will be measured using a 2 m flexible tape measure with buckle, around the midpoint between the iliac crest and inferior margin of the lower rib. Muscle mass will also be measured. Blood pressure will be measured at least 2/3rd of upper arm using appropriate sized cuff encircling, and at two times: initially after sitting quietly for 10 min, then again after 2 min using Mercury sphygmomanometer (model XJ1ID, China).
3. Health behavioral outcome measurements: The daily PA, screen media use, dietary behaviors and sleep health will be assessed.

The daily PA assessment including direct measures and indirect measures. For direct measures, daily PA will be measured using Actigraph wGT3X-BT accelerometers, which can provide objective as well as accurate measurement, and a valid and reliable estimate of PA in young people [15, 40]. Children will be instructed to wear the accelerometer on the non-dominant wrist for 7 days during waking hours (except during water-based activities) [41, 42] to measure duration, intensity and frequency of activity. To collected data of types of daily PA, self-administered Short form of International Physical Activity Questionnaire (IPAQ-A) will be used to subjectively measure daily PA. IPAQ-A is designed for large-scale research surveillance of adolescent PA [43]. Main questions in IPAQ-A assess PA habits by frequency; includes questions specific to school-related PA: during physical education, recess, at lunchtime, right after school, and evening and past weekend physical activities.

Additionally, questionnaires were designed to collect data about time of screen media use and dietary behaviors. Parents, guided by the study team, will report the number of minutes per day of screen media use (e.g., television, mobile electronic devices, video games and computers). Diet will be measured by a validated Dietary Screener Questionnaire (DSQ), a 26-item questionnaire which asks about the frequency of consumption in the past month of selected foods and drinks for children [44].

Assessment of sleep health will include both objective sleep parameters (total sleep time, sleep onset latency, sleep efficiency, and wake after sleep onset) and subjective parameters (sleep quality). Objective sleep parameters will be assessed using an accelerometer (Actigraph wGT3X-BT) for 7 consecutive days, 24 h/day. The Pittsburgh Sleep Quality Index (PSQI) was used to assess the subjective sleep quality and disturbances over a month time interval. It contains 18 items, generating seven scores, through the sum of score to assess the sleep quality. The higher the score, the worse the sleep quality [45].

### Project management and participant tracking

We developed an official WeChat account, and create 30 WeChat group. All parents of children download the app (WeChat) free of charge and create a unique identifying username.

Before the intervention starts, each WeChat team will be allocated a project staff from the study team. Under the direct leadership of the project director, project staffs will be responsible for:1) tracking team members to ensure that all necessary data are collected in a timely fashion; 2) providing timely and relevant feedback to the study team; and 3) sending education materials to the WeChat team.

### Statistical analyses

Descriptive statistics will be calculated using mean (SD) for continuous variables and n (%) for categorical variables. Differences will be compared using independent t-tests for continuous variables (e.g., blood sample outcomes) and Chi-squared tests for categorial variables (e.g., education and household income of parents). Linear mixed models will be used to test for group differences on the main outcome variables at follow-up visits, while subject ID will be random effect, the main effect include time, treatment group, and the interaction between time and treatment. We will include covariates that are not balanced between two groups into the model. Data will be entered and cleaned with EpiData 3.0, and managed and analyzed using SPSS 24.0 and SAS. Statistical significance will be set at *P* < 0.05.

## Discussion

An novel exercise intervention (‘S&G exercise intervention’) with WeChat as support, based on social incentives and gamification design, as well as ensuring intensity and dose of exercise, may have potential to improve metabolic health and even change unhealthy behaviors for long-term. In a randomized controlled trial design, the present study will assess the effectiveness of a ‘S&G exercise intervention’ to improve metabolic health and unhealthy behaviors.

The prevalence and severity of childhood obesity and metabolic abnormality in worldwide have increased [1, 31], which may lead to CVD and type 2 diabetes in adulthood [9, 10]. How to address it becomes an important demand worldwide. At present, several randomized controlled studies has proved that supervised exercise can improve metabolic risk factors, but once the supervised exercise is stopped, the health benefits weaken or vanish, and the exercise interventions also suffer from high-cost and small scale [46]. Remarkably, recent researches have suggested that social incentive and gamification were used in health interventions and have a potential effect on long-term maintenance [25, 47]. Moreover, WeChat also can provide an appropriate platform to deploy interventions on a broader, low-cost scale. Therefore, providing low cost, scalable and the long-term effective interventions to reduce metabolic risks among obese children becomes available.

To our knowledge, ‘S&G exercise intervention ’ was the first time to be designed, which make up traditional supervised exercise (SE) intervention limitation, and it can increase engagement as well as sustain exercise interventions effects on low cost, larger scale. Moreover, this study is not only targeted the effect of reduction of health outcomes including obesity and metabolic disorder, but also focused on healthy behavior changes including daily PA, diet pattern, sleep health and screen media use, which are significantly associated with health behaviors and health status in their adulthood. Hence, this study can also make a significant contribution to health research and practice by developing a new intervention model to promote unhealthy behaviors among obese children.

We predicted that this innovative exercise intervention (‘S&G exercise intervention package’) will develop improvements in metabolic health and unhealthy behaviors among obese children. We believed that this study could explore a low-cost, easy-to-popularize, and effective exercise intervention model for improving metabolic health and promote healthy among obese children. Furthermore, it will also provide important evidence for guidelines to prevent and improve metabolic health and health behaviors.

## Abbreviations

ADA: American Diabetes Association
BMI: Body mass index
CVD: Cardiovascular disease
DBP: Diastolic blood pressure
DSQ: Dietary Screener Questionnaire
FG: Fasting glucose
FI: Fasting insulin
HbA1c: Glycated (glycosylated) hemoglobin
HDL: High-density lipoprotein
HOMA-IR: Homeostatic Model Assessment for Insulin Resistance
HOMA-β: Homeostatic Model Assessment for β
IDF: International Diabetes Federation
IPAQ-A: Short form of International Physical Activity Questionnaire
PA: Physical activity
PSQI: The Pittsburgh Sleep Quality Index
SBP: Systolic blood pressure
SD: Standard deviation
SE: Supervised exercise
TG: Triglycerides
WGOC: Working Group on Obesity in China
WHO: World Health Organization
